# Developing a European grid infrastructure for cancer research: vision, architecture and services

**DOI:** 10.3332/ecms.2007.56

**Published:** 2007-09-21

**Authors:** M Tsiknakis, S Rueping, L Martin, S Sfakianakis, A Bucur, T Sengstag, M Brochhausen, J Pucaski, N Graf

**Affiliations:** 1 Biomedical Informatics Laboratory, Institute of Computer Science, Foundation for Research & Technology-Hellas, GR-71110 Heraklion, Crete, Greece; 2 Fraunhofer IAIS, Schloss Birlinghoven, 53754 St Augustin, Germany; 3 Biomedical Informatics Group, Artificial Intelligence Laboratory, School of Computer Science, Universidad Politécnica de Madrid, Spain; 4 Philips Research, Healthcare System Architecture, High Tech Campus 37, 5656 AE Eindhoven, The Netherlands; 5 Swiss Institute of Bioinformatics and Swiss Institute for Experimental Cancer Research, Bâtiment Génopode, CH-1015, Lausanne, Switzerland; 6 Institute for Formal Ontology and Medical Information Science (IFOMIS), Universität des Saarlandes, Postfach 151150, 66041 Saarbrücken, Germany; 7 Poznan Supercomputing and Networking Center, Noskowskiego 12/14, Poznan, Poland; 8 University Hospital of Saarland, Paediatric Haematology and Oncology, D-66421 Homburg, Germany

## Abstract

Life sciences are currently at the centre of an information revolution. The nature and amount of information now available opens up areas of research that were once in the realm of science fiction. During this information revolution, the data-gathering capabilities have greatly surpassed the data-analysis techniques. Data integration across heterogeneous data sources and data aggregation across different aspects of the biomedical spectrum, therefore, is at the centre of current biomedical and pharmaceutical R&D.

This paper reports on original results from the ACGT integrated project, focusing on the design and development of a European Biomedical Grid infrastructure in support of multi-centric, post-genomic clinical trials (CTs) on cancer. Post-genomic CTs use multi-level clinical and genomic data and advanced computational analysis and visualization tools to test hypotheses in trying to identify the molecular reasons for a disease and the stratification of patients in terms of treatment.

The paper provides a presentation of the needs of users involved in post-genomic CTs and presents indicative scenarios, which drive the requirements of the engineering phase of the project. Subsequently, the initial architecture specified by the project is presented, and its services are classified and discussed. A range of such key services, including the Master Ontology on sCancer, which lie at the heart of the integration architecture of the project, is presented. Special efforts have been taken to describe the methodological and technological framework of the project, enabling the creation of a legally compliant and trustworthy infrastructure. Finally, a short discussion of the forthcoming work is included, and the potential involvement of the cancer research community in further development or utilization of the infrastructure is described.

## Introduction

Life sciences are currently at the centre of an information revolution. Dramatic changes are being registered as a consequence of the development of techniques and tools that allow the collection of biological information at an unprecedented level of detail and in extremely large quantities. Advanced technologies, such as high-throughput screening, genomics, proteomics and metabonomics, have resulted in data generation on a previously unknown scale.

The nature and amount of information now available opens up areas of research that were once in the realm of science fiction. Pharmacogenomics [1], diagnostics [2] and drug target identification [3] are just a few of the many areas that have the potential to use this information to change dramatically the scientific landscape in the life sciences.

During this information revolution, the data-gathering capabilities have greatly surpassed the data-analysis techniques. If we were to imagine the Holy Grail of life sciences, we might envision a technology that would allow us to fully understand the data at the speed at which these are collected. Sequencing, localization of new genes, functional assignment, pathway elucidation and understanding the regulatory mechanisms of the cell and organism should be easy. Ideally, we would like knowledge manipulation to become as efficient as goods manufacturing is today: highly automated, producing more goods, of higher quality and in more cost-effective manner than manual production. It is our belief that, in a sense, knowledge manipulation is now reaching its pre-industrial age. The explosive growth in the number of new and powerful technologies within proteomics and functional genomics can now produce massive amounts of data, but using it to manufacture highly processed pieces of knowledge still requires skilled elaborate involvement of experts to forge through small pieces of raw data one at a time. The ultimate challenge in the coming years, we believe, will be to automate this knowledge discovery process.

Therefore, data integration across heterogeneous data sources and data aggregation across different aspects of the biomedical spectrum is at the centre of current biopharmaceutical R&D. A technological infrastructure supporting such a knowledge discovery process should, ideally, allow for:

1. Data to be searched, queried, extracted, integrated and shared in a scientifically and semantically consistent manner across heterogeneous sources, both public and proprietary, ranging from chemical structures and omics to clinical trials data.
2. Discovery and invocation of scientific tools that are shared by the community, rather than repeatedly developed by each and every organization that needs to analyse their data.
3. Apart from the sharing of tools, it should also allow for their integration as modules in a generic framework and applied to relevant dynamic datasets. We refer to this process as ‘discovery driven scientific workflows’, which ideally would also be executed in a fast, unsupervised manner.

**Figure 1:**
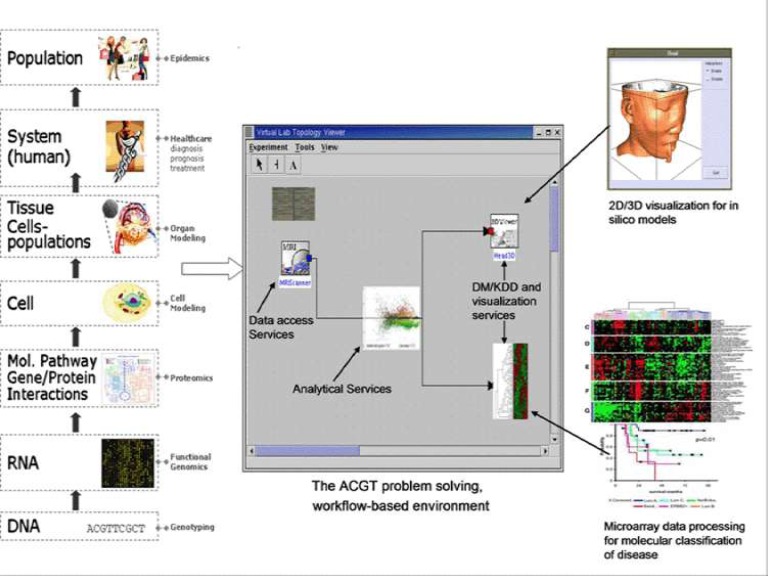
The envisaged ACGT problem-solving environment supporting integrated access and analysis of multi-level, heterogeneous and distributed biomedical data.

Needless to say, our current inability to efficiently share data and tools in a secure and efficient way is severely hampering the research process. The objective of the Advancing Clinico-Genomic Trials on Cancer (ACGT) Project is to contribute to the resolution of these problems through the development of a unified technological infrastructure, which will facilitate the easy and secure access and analysis, of multi-level clinical and genomic data enriched with high-performing knowledge discovery operations and services in support of multi-centric, post-genomic clinical trials (see Figure 1).

This paper presents a short background section discussing the urgent needs faced by the biomedical informatics research community, and very briefly describes the clinical trials upon which the ACGT project is based for both gathering and eliciting requirements and also for validating the technological infrastructure designed. It continues with a presentation of the initial ACGT architecture and presents its layers and key enabling services. The rest of the paper is focused on the functional description of key services that jointly create the integrated technological infrastructure of ACGT, supporting easy data integration and knowledge discovery. The final section critically discusses open issues and particularly focuses on the issue of community involvement. It presents ways in which such involvement may be fostered by the project.

## Post-genomic clinical trials on cancer

In ACGT, we focus on the domain of clinical trials on cancer. It is well established that patient recruitment is often the time-limiting factor for clinical trials. As a result, clinical trials are gradually turning multi-centric to limit the time required for their execution [4].

Also, with respect to cancer research, the use of high-throughput technologies has resulted in an explosion of information and knowledge about cancers and their treatment. Cancer, being a complex multi-factorial disease group that affects a significant proportion of the population worldwide, is a prime target for focused multi-disciplinary efforts using these novel and powerful technologies [2]. Exciting new research on the molecular mechanisms that control cell growth and differentiation has resulted in a quantum leap in our understanding of the fundamental nature of cancer cells.

While these opportunities exist, the lack of a common infrastructure has prevented clinical research institutions from being able to mine and analyse disparate, multi-level data sources. As a result, very few cross-site studies and multi-centric clinical trials are performed, and in most cases, it is not possible to easily integrate multi-level data (from the molecular to the organ and individual levels).

The ACGT project has been structured within such a context. It has selected two cancer domains and has defined three specific trials. These trials serve a dual purpose. Firstly, they are used for developing a range of post-genomic analytical scenarios to feed the requirement analysis and elicitation phase of the project, and secondly, they will be used for the validation of the functionality of the ACGT technologies.

The ACGT trials are in the domain of breast cancer and Wilm’s tumour (pediatric nephroblastoma). Specifically:

1. The ACGT test of principle (TOP) study aims to identify biological markers associated with pathological complete response to anthracycline therapy (epirubicin), one of the most active drugs used in breast cancer treatment [5].
2. Wilms’ tumour, although rare, is the most common primary renal malignancy in children and is associated with a number of congenital anomalies and documented syndromes.

The goal of the current clinical trial is to reduce therapy for children with low-risk tumours, thereby avoiding acute and long-term toxicities. Challenges remain in identifying novel molecular, histological and clinical risk factors for stratification of treatment intensity. This could allow a safe reduction in therapy for patients known to have an excellent chance of cure with the current therapy, while identifying, at diagnosis, the minority of children at risk of relapse, who will require more aggressive treatments [6].

In addition to these trials, and on the basis of data collected for the purpose of their execution, an *in silico* modelling and simulation experiment is also planned. The aim of this experiment is to provide clinicians with a decision support tool able to simulate, within defined reliability limits, the response of a solid tumour to therapeutic interventions based on the individual patient’s multi-level data. The most critical biological phenomena (e.g. metabolism, cell cycling, geometrical growth or shrinkage of the tumour, cell survival, following irradiation or chemotherapeutic treatment, necrosis, apoptosis) will be thus spatiotemporally simulated using a variety of clinical, radiobiological, pharmacodynamic, molecular and imaging data [7].

For a more elaborate description of these trials, readers are referred to [8].

### Technical challenges

**Figure 2:**
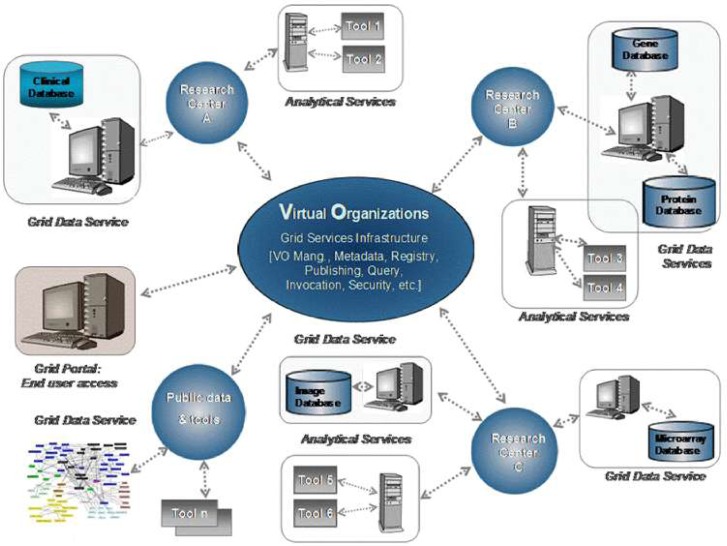
The vision of ACGT. Creating and managing virtual organizations on the grid who are jointly participating in the execution of clinical trials and who decide to adopt the principles of sharing of both data and tools.

The ACGT’s vision is to become a pan-European voluntary network connecting individuals and institutions and to enable the sharing of data and tools (see Figure 2). In order to achieve its goals and objectives, ACGT is creating an infrastructure for cancer research by using a virtual web of trusted and interconnected organizations and individuals to leverage the combined strengths of cancer centres and investigators and enable the sharing of biomedical cancer-related data and research tools in such a way that the common needs of interdisciplinary research are met and tackled.

Considering the current size of clinical trials (hundreds of patients), there is a clear need, both from the viewpoint of the fundamental research and from that of the treatment of individual patients, for a data analysis environment that allows the exploitation of this enormous pool of data [9].

A major part of the project is devoted to research and development in infrastructure components that are gradually being integrated into a workable demonstration platform upon which the selected (and those to be selected during the lifecycle of the project) clinical studies will be demonstrated and evaluated against user requirements defined at the onset of the project.

### Scientific and functional requirements

The real and specific problem that underlies the ACGT concept is coordinated resource sharing and problem solving in dynamic, multi-institutional, pan-European virtual organizations. A set of individuals and/or organizations defined by such sharing relationships form what we call ‘an ACGT virtual organization’ (VO) [10]. Simply stated, the participants in a multi-centric clinical trial form a VO, which exists for the duration of a trial or for any other period of time based on mutual agreements.

The task, therefore, of ACGT is to make data and tools securely available in this inter-enterprise environment, where and when needed, to all authorized users. As a result, the scientific and functional requirements for the ACGT platform can be summarized as follows:

1. Virtual organization management: support for the dynamic creation of VOs, defined as a group of individuals or institutions who share the computing and other resources of a ‘grid’ for a common goal.
2. Data federation: seamless navigation across and access to heterogeneous data sources, both private and public.
3. Data integration: the capacity to pool data from heterogeneous sources in a scientifically, semantically and mathematically consistent manner for further computation.
4. Shared services: the development, sharing and integration of relevant and powerful data exploitation tools such as tools for bioinformatics analysis, data mining, modelling and simulation.

The requirements for the elicitation process that has taken place in the project, based on input for a diverse range of users, has resulted in the identification of the following key technical requirements:

1. Flexibility: in other words, modularity (supporting integration of new resources in a standardized way) and configurability (accommodating existing and emerging needs). This is required because (a) The a priori scientific and functional requirements are broad and diverse; (b) the data resources to be federated by the ACGT platform are characterized by deep heterogeneities in terms of source, ownership, availability, content, database design, data organization, semantics and so on; and (c) the complexity of the underlying science, as well as the complexity of applicable knowledge representation schemas and applicable scientific algorithms.
2. Intuitive access to information: from the user’s point of view, the ACGT knowledge management platform must provide relevant and simple access to information—both in terms of searching and navigation—and to services. In addition, it must provide a dynamically evolving set of validated data exploration, analysis, simulation and modelling services.
3. Security: finally, it must be consistent with the European ethical and legal framework, providing a high degree of trust and security to its users.

#### Evaluation scenarios

We have adopted a scenario-based development process. A range of scenarios, that is the tasks users want to perform, structured and described as a sequence of activities that require access to heterogeneous data, use of various tools for its analysis and invocation of appropriate tools for visualizing and interpreting the results, have been defined by the ACGT user community.

These scenarios cover the most important functional goals of the infrastructure. In practice, the following fields are covered:

1. Administrative scenarios related to the setup and maintenance of the infrastructure, such as integration of databases.
2. Administrative scenarios related to the management of users and institutions in the context of virtual organizations.
3. Technological scenarios, validating the integration of data analysis tools per se (e.g. R) and their integration with clinical data.
4. Clinical-oriented scenarios, validating the analysis tools as used by clinicians and biomedical researchers in realistic contexts.
5. Meta-analysis scenarios, validating the use of ACGT as clinical-research validation tool.

Space does not allow for a detailed description of these scenarios. Interested readers should access the project website, where relevant information will be available soon in the project’s quarterly Newsletter.

### The ACGT architecture and services

In principle, the requirements for the ACGT platform can be met by designing a federated environment articulating independent tools, components and resources based on open architectural standards, which is customizable and capable of dynamic reconfiguration.

Considering that the amount of data generated in the context of post-genomic clinical trials is expected to rise to several gigabytes of data per patient in the near future, access to high-performance computing resources will be unavoidable. Hence, grid computing [11] appears to be a promising technology. Access and use of grid-based resources is thus an integral part of the design of the infrastructure.

A layered approach has been selected for providing different levels of abstraction and a classification of functionality into groups of homologous software entities [12]. In this approach, we consider the security services and components to be pervasive throughout ACGT so as to provide both for the user management, access rights management and enforcement, and trust bindings that are facilitated by the grid and domain-specific security requirements like pseudonymization and anonymization.

In specifying the initial architecture of the ACGT technological platform, architectural specifications of other relevant projects have been thoroughly studied. Of particular relevance are the Cancer Biomedical Informatics Grid (caBIG) [13] in the United States and the CancerGrid [14] Project in the United Kingdom.

The overall approach in these projects is somewhat different from the one in ACGT. In caBIG, the bottom-up, technology-oriented approach was chosen, in which the focus was put on the integration of a large number of analytical tools but with weak concern on data-privacy issues. CancerGrid on the other hand addresses the specific needs of the British clinical community. As a result, some aspects of the project may not fully overlap with the European and international scope of ACGT.

**Figure 3:**
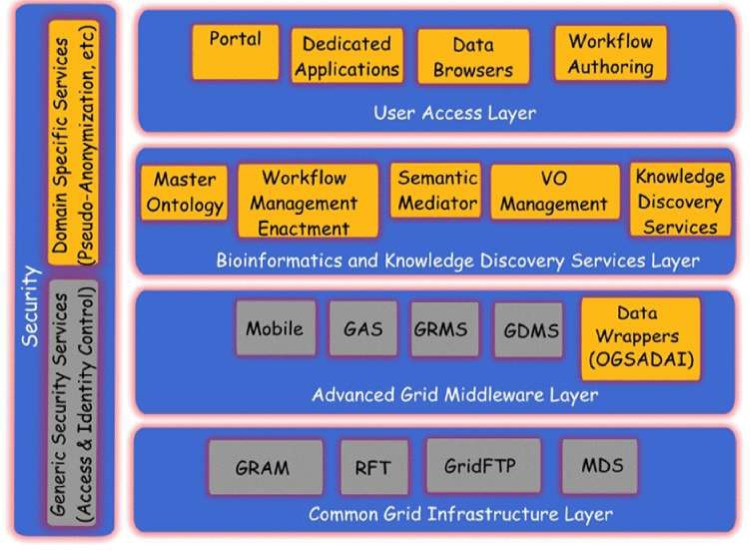
The ACGT layered architecture and its main services.

The ACGT environment is designed to be versatile and will allow the integration of high-throughput databases with data both from existing (e.g. microarrays, imaging) and future technologies (e.g. high-throughput proteomics). The design of the platform considers the integration of private (i.e. trial-specific) databases with public ones, thus potentially making public datasets immediately available for hypothesis validation and meta-analyses. An overview of the ACGT system layered architecture is given in Figure 3. The various layers of the architecture are briefly described in the following. For a more detailed description, the reader is referred to [9, 12].

**Figure 4:**
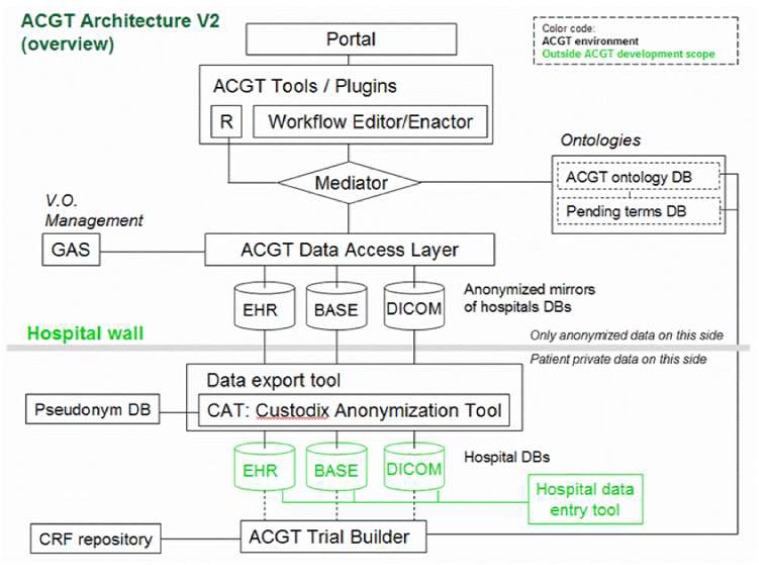
Simplified ACGT architecture assumed in the context of the present document. Data are made available for federated access to an ACGT virtual organization, following anonymization (CAT tool); data access is only permitted to authorized members of an ACGT virtual organization through the use of appropriate services (GAS—Gridge Authorisation Service).

Of particular importance is the security layer of the architecture; access rights, security (encryption) and trust building are issues addressed and solved on this layer, based on system architectural and security analysis [15]. Also, domain-specific security services, such as pseudo-anonymization and anonymization services (CAT in Figure 4) are modelled and invoked through this layer (see section ‘The ACGT security Framework’).

In subsequent sections we describe, in slightly more details, the Bioinformatics and Knowledge Discovery Services, which represent the ‘workhorse’ of ACGT and the corresponding layer is the place where the majority of ACGT specific services lie. Some characteristic examples of such key services are the (a) Mediator Services that offer uniform access to distributed and heterogeneous clinical trial and other post-genomic biomedical databases; (b) Ontology Services that provide a conceptualisation of the domain through the Master Ontology on Cancer and the ‘domain of discourse’ for constructing complex queries for the mediator services; (c) Workflow Enactment Services that support the efficient management and execution of complex biomedical workflows; (d) Metadata Repositories and the corresponding services for the persistent management of services’ metadata descriptions; and (e) an assortment of data mining and knowledge discovery tools and services that fulfil the data analysis requirements of ACGT.

#### Validation of the initial architecture

Anticipated scenarios for the evaluation and validation depend heavily on the data flow inside the ACGT infrastructure, which in turn depend on the architecture retained. Figure 4 provides a less abstract (and somewhat simplified and hence easier to be understood by non-experts) description of the reference architecture.

One important feature of this architecture is the isolation of the patients’ private information from the core of the ACGT environment described in the initial DoW; only properly anonymized data are allowed to flow outside the hospital (in the present context ‘hospital‘ refers to any institution legally housing the database containing identifiable patient information.

In order to facilitate data access and implementation of legacy code in the ACGT environment without rewriting interfaces, an anonymized mirror of the clinical-trial databases is maintained. It is the latter that will be accessible to clinicians and researchers, once properly authenticated.

### Key bioinformatics and knowledge discovery services

#### Heterogeneous biomedical database integration

Distributed and heterogeneous databases, created in the context of multi-centric, post-genomic clinical trials on cancer, need to be easily accessible and transparently queried in the context of a user’s discovery driven analytical tasks. A central challenge, therefore, to which ACGT needs to respond, is the issue of semantic integration of heterogeneous biomedical databases.

The process of heterogeneous database integration may be defined as ‘the creation of a single, uniform query interface to data that are collected and stored in multiple, heterogeneous databases’. Several varieties of heterogeneous database integration are useful in biomedicine. The most important ones are:

1. *Vertical integration:* the aggregation of semantically similar data from multiple heterogeneous sources. For example, a ‘virtual repository’ that provides homogeneous access to clinical data that are stored and managed in databases across a regional health information network is reported in [16, 17, 18].
2. *Horizontal integration:* the composition of semantically complementary data from multiple heterogeneous sources. For example, systems that support complex queries across genomic, proteomic and clinical information sources for molecular biologists are reported in [19, 20, 21].

Typical examples of research projects addressing the issue of heterogeneous biomedical database integration are the TAMBIS (Transparent Access to Multiple Bioinformatics Information Sources) [19] Project, which creates a bioinformatics domain ontology by using the GRAIL Description Logic Language. Mapping concepts to existing information sources, queries against the ontology are evaluated by accessing individual sources in a user-transparent manner. Although this approach is novel, scalability and expressivity are a concern. Also, the depth and quality of the TAMBIS ontology, or its overlap with existing biomedical ontologies, such as the gene ontology, are difficult to evaluate because the ontology contents are not contributed currently to a source, such as UMLS or other.

Also, an information mediator prototype, called KIND (knowledge-based integration of neuroscience data) [21], has been developed as part of an integrated neuroscience workbench project at SDSC/UCSD within the NPACI project (National Partnership for Advanced Computational Infrastructure, http://www.npaci.edu.) The broad goal of the workbench is to serve as an environment where, among other tasks, the neuroscientist can query a mediator to retrieve information from across a number of information sources, and use the results to perform her own analysis on the data.

In ACGT, we have adopted an ontology-based approach to the integration challenge [22]. As a result, two services become of paramount importance in our architecture; the Master Ontology on Cancer and the Mediator Service. These are briefly discussed in the next sections.

#### The data-access architecture

**Figure 5:**
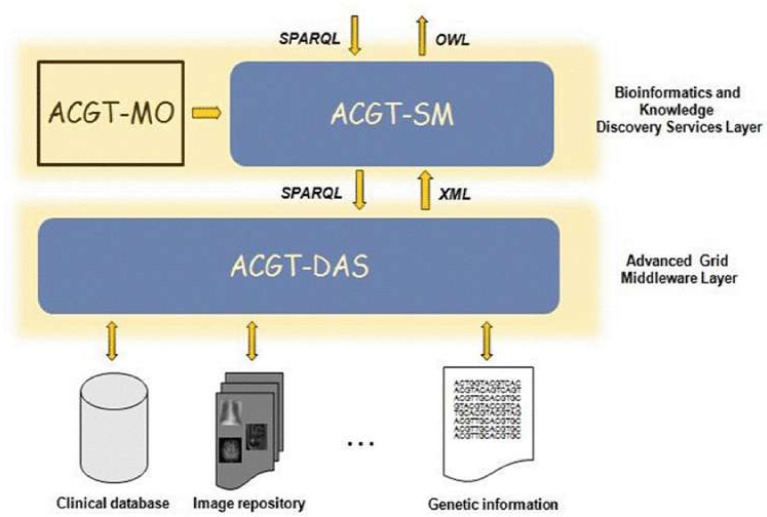
The data-access architecture.

As said previously, the ACGT data-access architecture is composed of a set of key services, namely the ACGT-DAS, the ACGT-SM, the ACGT-MO and some additional dedicated tools. While the first two services provide the means to resolve syntactic and semantic heterogeneities when accessing heterogeneous databases, the latter acts as a core resource supporting the data-integration process. Figure 5 presents the detailed data-access architecture in ACGT, showing the interactions between these three main services.

The following sections explain in more detail the various issues faced in the implementation of the ACGT-MO and the ACGT-DAS and the ACGT-SM services.

#### The ACGT Master Ontology (MO) on Cancer

The ACGT Consortium chose ontologies as the main knowledge representation (KR) tool in order to represent the relevant parts of medical knowledge gathered along the years by cancer researchers and clinicians involved with the theory and practice of oncology. We will not spend much time here debating the advantages of ontologies versus other KR strategies, since we trust that this has been covered elsewhere in the relevant literature [23, 24, 25]. We simply believe that biomedicine is one of the research fields that stands to benefit greatly from the ‘ontological turn’, as can be seen from the ACGT project.

Among the challenges of the ACGT MO development, the large scope of the project was certainly the most demanding. Many areas, such as clinical studies, clinical cancer management and care, genomic research, etc, had to be reflected; all these, on the other hand, could easily make the subject of a plethora of more focused and targeted domain ontologies, wherefrom, ideally, the ACGT MO might be constructed in a modular manner. That, unfortunately, could not happen, be it for the simple fact that no such targeted ontologies exist yet, or are not in a consistent shape to meet the quality demands of the ACGT consortium. ACGT-partner IFOMIS (http://www.ifomis.uniaarland.de/) is active in numerous international efforts aimed at developing cutting-edge ontologies. The ontology for biomedical investigation (OBI), for example is an ontology that ‘will support the consistent annotation of biomedical investigations, regardless of the particular field of study’ [26].

In particular, and quite remarkably, the section of the OBI dubbed the ‘ontology of clinical investigation—OCI’ (http://groups.google.com/group/OCInv) turns out to be covering part of the domain presently embraced by the ACGT MO. Given that ACGT researchers deal, at the present time, on both fronts, we expect not mere compatibility, but a great deal of integration and convergence between these parallel efforts, to the point where, upon attaining a reasonable degree of stability, we envisage importing the OCI (as well as some other relevant branches of the OBI) into the ACGT MO. The modular character of MO is also reflected via the use of FMA [27] and GO [28]. We, nevertheless, still like to regard the ACGT MO as a single-domain ontology, as this is likely to guarantee a uniform treatment of the issues covered by the ACGT umbrella.

In order to provide a consistent and sound representation, the ACGT MO employs the resources of a top-level ontology or upper-level ontology, which is, according to the Standard Upper Level Ontology Working Group of IEEE, ‘limited to concepts that are meta, generic, abstract and philosophical and therefore are general enough to address (at a high level) a broad range of domain areas. Concepts specific to given domains will not be included; however, this standard will provide a structure and a set of general concepts upon which domain ontologies (e.g. medical, financial, engineering) could be constructed’ [29]. We have chosen the Basic Formal Ontology (BFO) (http://www.ifomis.uni-saarland.de/bfo/) as top level for the ACGT MO, since BFO has proven to be highly applicable to the biomedical domain [30]. The ACGT Master Ontology, hence, inherits BFO’s foundational principles: realism (ontologies as representations of reality rather than concrete specifications of conceptual schemes), perspectivalism (many equally valid perspectives on reality), fallibilism (our ontologies are fallible and perpetually evolving), and adequatism (no emphasis on reducing the various ontological categories to few basic ones) (readers are referred to the BFO manual [31] for detailed explanations).

The ACGT MO is presented as an .owl file and is written in OWL-DL. It was built, and is being maintained/curated, using the Protégé-OWL free open-source ontology editor (http://protege.stanford.edu/).

The process that gave rise to the present state of the representation of clinical reality was rather convoluted and highly elaborate, requiring multiple recurring steps and a multifaceted approach. Firstly, actual case report forms (CRFs) from ACGT trials were collected and analysed with respect to the universals (classes) more-or-less explicitly present in the information gathered. In parallel, basic aspects of cancer pathology and cancer management were studied by our researchers. The outcome of these activities provided the basic information on the universals and relations (properties) captured in the ACGT MO. This ontology prototype was made available to all partners in the project. In addition, clinical partners were asked to review the prototype with respect to clinical accuracy and technical partners for reviews on the usability. Based on the results of these reviews, the ontology was refined step by step, keeping up the collaboration with all partners in the consortium and asking for their constant review of results.

#### The data-access services (ACGT-DAS)

The data-access layer (shown in Figure 4) is implemented through the appropriate services, the data-access services (ACGT-DAS in Figure 5). They provide a uniform data-access interface. This includes uniformity of transport protocol, message syntax, query language and data format. They are also used for exporting the structure of the database, using a common data model, together with possible query limitations of the data source. Finally, since strict legal and ethical requirements with respect to data access exist that need to be adhered to, they enforce the data source access policy, and audit access to data sources. For the first implementation, we focused our effort on the first requirement, so that integration with the ACGT-SM, using test databases, could start as soon as possible.

Web services have been chosen as the common interface technology within ACGT, as this technology suits the distributed nature of the project with respect to the data, computing resources and development teams. More specifically, the ACGT-DAS are implemented as OGSA-DAI services, a Web services framework for data access [32], which uses an activity framework that enables flexible service invocation and reuse of common data-access functionality.

We have implemented data-access services for two data source types: relational databases and medical image databases. These data sources have the benefit that there are established standards for data access, namely SQL/JDBC for relational databases, and DICOM for medical image databases.

Currently, appropriate services are implemented for accessing other databases managing post-genomic data (metabolomics, proteomics, etc) as well as public biomedical databases.

#### The semantic mediator (ACGT-SM)

The semantic mediation process is realized by a set of tools that support the database integration process. The core component is the ACGT-SM, in charge of exposing the global schema, processing queries and retrieving integrated results. Its functionality is offered as a Web service to other components, that is knowledge discovery tools, the workflow editor, other specific end-user tools, etc. Additional components that are used for overcoming several issues in the data integration process, as discussed previously, are the mapping tool, the data cleaning module—for retrieved instances—and the query preprocessing module, for literal homogenization in queries. Technical description of the role and functions of these tools goes beyond the scope of the current paper. It suffices to say that the complex ‘semantic data integration’ tasks are addressed in the ACGT architecture through a family of interoperating generic and specific tools and services.

A case study, based on the integration of two clinical trial databases, that is the SIOP and the TOP databases, filled with test data (actual patient data were avoided due to privacy issues) and a corresponding DICOM image database was performed. The sources were successfully integrated, and the schemas, that is the views representing the underlying databases, produced after the integration process, were validated by domain experts. This case study is fully documented in [33] and experiences are discussed in the following section.

#### Knowledge discovery services

Once these multi-level clinical and genomic data are integrated, they can be mined to extract new knowledge that can be useful in topics such clinical diagnosis, therapy, prevention and, of course, the design of new studies (such as in the case of ACGT, clinico-genomic trials).

Knowledge discovery in clinico-genomic data presents a new array of challenges since it differs significantly from the original problems of data analysis that prompted the development of grid technologies, for example in particle physics and astronomy [9, 34]. The exploitation of semantics information in the description of data sources and data analysis tools is of high importance for the effective design and realization of knowledge discovery processes. Semantics are usually made concrete by the adoption of metadata descriptions and relevant vocabularies, classifications and ontologies. In ACGT, these semantics descriptions are managed by the grid infrastructure, and therefore the knowledge discovery services build and operate on a knowledge grid platform [35].

#### Workflows

The Workflow Management Coalition (WFMC, http://www.wfmc.org/) defines a workflow as ‘The automation of a business process, in whole or part, during which documents, information or tasks are passed from one participant to another for action, according to a set of procedural rules’. In other words, a workflow consists of all the steps and the orchestration of a set of activities that should be executed in order to deliver an output or achieve a larger and sophisticated goal. In essence, a workflow can be abstracted as a composite service, that is a service that is composed of other services that are orchestrated in order to perform some higher level functionality.

The aim of the ACGT workflow environment is to assist the users in their scientific research by supporting the ad hoc composition of different data access and knowledge extraction and analytical services into complex workflows. This way the users can extend and enrich the functionality of the ACGT system by reusing existing ACGT compliant services and producing ‘added value’ composite services. This reuse and composition of services is in some sense a programming task where the user actually writes a program to realize a scenario or to test a scientific hypothesis.

**Figure 6:**
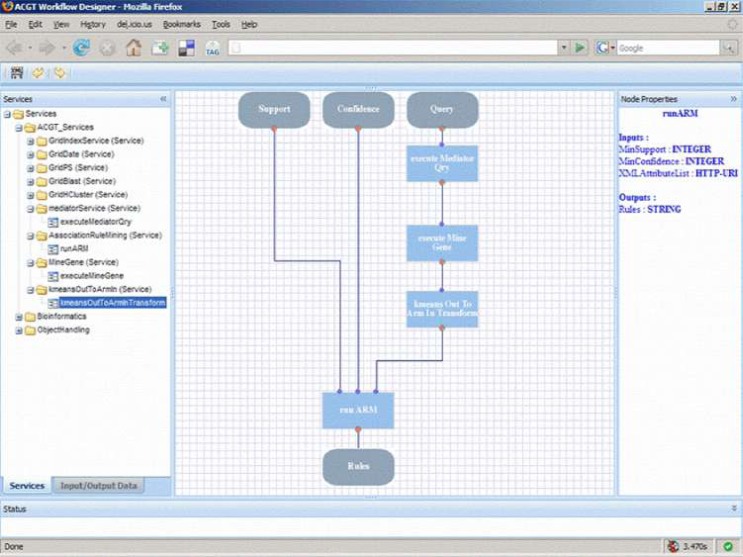
A typical layout of the ACGT workflow designer that allows users, through the ACGT portal, to design and execute their scientific explorations by seamlessly integrating the various data access services and analytical tools in what we refer to as ‘discovery workflows’.

In order to support the ACGT users to build and design their workflows, a visual workflow-programming environment has been designed (Figure 6). It is a Web-based workflow editor and designer that is integrated into the rest of ACGT system so as to take advantage of the grid platform and the ACGT specific infrastructure and services. In particular, this workflow designer features a user-friendly graphical user interface (GUI) that supports the efficient browsing and searching of the available ACGT services and their graphical interconnection and manipulation to construct complex scientific workflows. The choice of a graphical representation of the workflow and the support for ‘point-and-click’ handling of the workflow graph was made on the basis that this is more intuitive for the users and increases their productivity. Additional features that also take advantage of the metadata descriptions of services include the validation in the design phase of the workflows in order to reduce or even eliminate the incorrect combination of processing units and the provision of a ‘service recommendation’ functionality based on the data types and data formats of inputs and outputs and are currently under development.

The architecture of the workflow environment also includes a server side component for the actual execution (‘enactment’) of workflows. Each workflow is deployed as a ‘higher order’, composite service and the workflow enactor is the grid-enabled component responsible for the invocation, monitoring and management of running workflows. The standard workflow description language WS-BPEL [36] has been selected as the workflow description format and being a standard it enables the separation of the workflow designer from the workflow enactor and facilitates their communication and integration: the designer is a ‘rich internet application’ running inside the users’ browsers that stores the workflows in WS-BPEL format into a workflow-specific repository, whereas the enactor is an ACGT service running into the ACGT grid that ‘revitalizes’ the persisted workflows as new services.

##### Data, service and workflow metadata

Easy integration of applications and services requires substantial meta-information on algorithms and input/output formats if tools are supposed to interoperate. Furthermore, assembly of tools into complex ‘discovery workflows’ will only be possible if data formats are compatible and semantic relationships between objects shared or transferred in workflows are clear. In achieving such requirements, the use of meta-data is important. As a result, in ACGT we focus on the systematic adoption of metadata to describe grid resources, to enhance and automate service discovery and negotiation, application composition, information extraction and knowledge discovery [37]. Metadata is used in order to specify the concrete descriptions of things. These descriptions aim to give details about the nature, intent, behaviour, etc, of the described entity, but they are also data that can be managed in the typical ways so this explains the frequently used definition: ‘metadata are data about data’.

Examples of this data may be research groups, participating in a CT and publishing the datasets, data types that are being exposed, analytical tools that are published, the input data format required by these tools and the output data produced and so forth. Some of types of metadata that have been identified are as follows:

1. Contact info: contact info and other administrative data about a site participating in a CT who shares information on the grid.
2. Data type: the data type that a site is exposing and the context upon which this data were generated.
3. Data collection method: this would include the name of the technique or the platform that was used to perform the analysis (e.g. Affymetrix), its model and software version, etc.
4. Ontological category: an ontological category describes a particular concept that the dataset exposes or a tool operates upon.

#### Analytical services metadata

Similarly, the identified analytical services’ metadata descriptions fall into the following categories:

1. the *task* performed by the service; that is the typology of the analytical data analysis process (e.g. feature/gene selection, sample/patient categorization, survival analysis);
2. the *steps* composing the task and the order in which the steps should be executed;
3. the *method* used to perform an analytical/bioinformatics task;
4. the *algorithm* implemented by the service;
5. the *input data* on which the service works;
6. the kind of *output* produced by the service.

Our ultimate challenge is to achieve the implementation of semantically aware grid services. In achieving this objective, a service ontology is being developed to provide a single point of reference for these concepts and to support reasoning of concept expressions.

### Creating and sharing ACGT compliant services

Achieving the level of automation, that is graphically depicted in Figure 6, requires the creation of highly interoperable services. Creating a service involves describing, in some conventional manner, the operations that the service supports; defining the protocol used to invoke these operations over the internet and operating a server to process incoming requests.

Although a fair amount of experience has been gained with the creation of services and applications in different science domains, significant problems do still remain, especially with respect to interoperability, quality control and performance. These are issues to which ACGT focuses, and these are briefly discussed in the next subsections.

#### Interoperability and reuse

Services have little value if others cannot discover, access and make sense of them. Yet, as Stein has observed [38], today’s scientific communities too often resemble medieval Italy’s collection of warring city, states, each with its own legal system and dialect. Available technological (i.e. Web services) mechanisms for describing, discovering, accessing and securing services provide a common alphabet, but a true lingua franca requires agreement on protocols, data formats and ultimately semantics. In the ACGT project, we are paying particular attention on these issues and especially on the issue of semantics (see section on metadata).

#### Management

In a networked world, any useful service will become overloaded. Thus, we need to control who uses services and for what purposes. Particularly, valuable services may become community resources requiring coordinated management. Grid architectures and software—a set of Web services technologies focused on distributed system management—can play an important role in this regard [39], and ACGT is focusing on exploiting these opportunities made available by grid computing.

#### Quality control

As the number and variety of services grow and interdependencies among services increase, it becomes important to automate previously manual quality control processes—so that, for example users can determine the provenance of a particular derived data product [40]. The ability to associate metadata with data and services can be important, as can the ability to determine the identity of entities that assert metadata, so that consumers can make their own decisions concerning quality.

### The ACGT security framework

We recognise that the sharing of multi-level data outside the walls of a hospital or a research organization generates complex ethical and legal issues. It is also well known that the concerns around ‘security issues’ have been one of the major obstacles that have inhibited wider adoption of information technology solutions in the healthcare domain. As a result, we have devoted significant efforts in the study and analysis of the ethical and legal issues related to cross-institutional sharing of post-genomic datasets and have defined every aspect, both technical and procedural, of the required security framework. It is worth mentioning at this stage that security and privacy are active areas of research, and technologies are emerging that are fully utilized in ensuring the highest possible level of security of the ACGT platform.

Based on such an approach we concluded that trust and security must to be addressed at multiple levels; these include (a) infrastructure, (b) application access, (c) data protection, (d) access control, which would be policy governed and (e) privacy-enhancing technology, such as de-identification.

**Figure 7:**
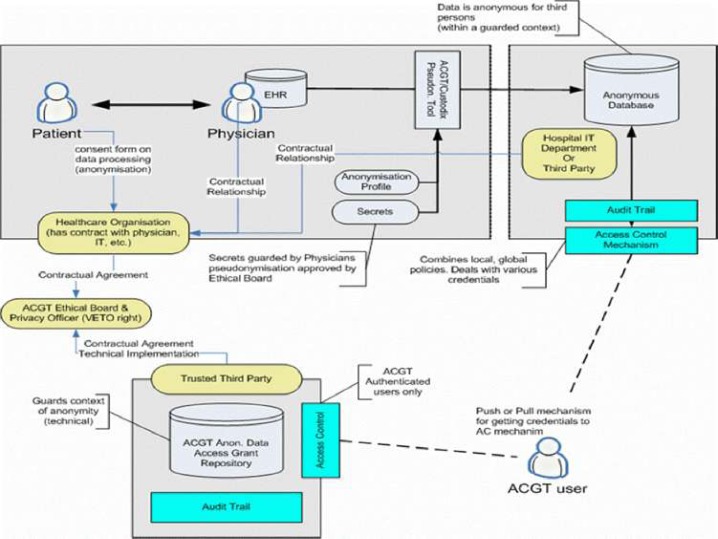
Overview of the ACGT data protection framework: actors and interactions.

The European Directive on Data Protection [41] deals with the protection of personal data and imposes many restrictions on its use. In order to allow ACGT partners to handle and exchange medical data in conformance with the requirements of European Directive on Data Protection, an advanced data protection framework has been designed. This framework (illustrated in Figure 7) achieves this goal through an integrated approach that includes technical requirements but also policies and procedures (for more details see [8], Post-genomic clinical trials—the perspective of ACGT, in the same issue of this journal). Some of the aspects of the data protection framework are (a) anonymization or pseudonymzation of the data, (b) a trusted third party (TTP) pseudonymization and a corresponding pseudonymization tool, (c) technology-supported measures to control the anonymity context, (d) an ACGT data protection board (acting as a TTP) responsible for issuing credentials for data access to authorized users and (e) definition of the necessary consent forms and legal agreements that need to be signed by all members of any ACGT Virtual Organization.

Description of the technical details of the security architecture of ACGT (the data protection framework) goes beyond the scope of the current article. Nevertheless, the main message that we want to stress is the fact that a well designed set of both technological as well as procedural measures have been taken, so that a high degree of trust and security is built into the final infrastructure to be delivered.

### Discussion and outlook

In this paper, we consider a world where biomedical software modules and data can be detected and composed to define problem-dependent applications. We wish to provide an environment allowing clinical and biomedical researchers to search and compose bioinformatics and other analytical software tools for solving biomedical problems. We focus on semantic modelling of the requirements of such applications using ontologies.

The objective of this article is not so much to describe in detail the technological aspects of the infrastructure being developed. Rather, our objective is to present the vision behind our work, to reveal the anticipated benefits for cancer research and to present the main technological challenges we are addressing. At the same time, we seek to interact, at this early stage of our implementation plan, with the widest possible community of our future users with the objective of crystallizing requirements and ensuring that we are indeed responding to real user needs.

The project has conceived an overall architecture for an integrated biomedical sciences platform. The infrastructure being developed uses a common set of services and service registrations for the entire clinical trial on cancer community. We are currently focusing on the development of the core set of components up to a stage where they can effectively support *in silico* investigation. Initial prototypes have been useful in crystallizing requirements for semantics.

The project has set up cross-disciplinary task forces to propose guidelines concerning issues related to data sharing, for example legal, regulatory, ethical and intellectual property, and is developing enhanced standards for data protection in a Web (grid) services environment.

In addition, the project is developing:

1. standards and models for exposing Web services (semantics), scientific services, and the properties of data sources, datasets, scientific objects, and data elements;
2. new, domain-specific ontologies, built on established theoretical foundations and taking into account current initiatives, existing standard data representation models and reference ontologies;
3. innovative and powerful data exploitation tools, for example multi-scale modelling and simulation, considering and integrating from the molecular to the systems biology level and from the organ to the living organism level;
4. standards for exposing the properties of local sources in a federated environment;
5. a biomedical grid infrastructure offering mediation services for sharing data and data-processing methods and tools;
6. advanced security tools including anonymization and pseudonymization of personal data according to European legal and ethical regulations;
7. a Master Ontology on Cancer and use of standard clinical and genomic ontologies and metadata for the semantic integration of heterogeneous databases;
8. an ontology-based trial builder for helping to easily set up new clinico-genomic trials, to collect clinical, research and administrative data and to put researchers in the position to perform cross-trial analysis;
9. data-mining services in order to support and improve complex knowledge discovery processes;
10. an easy-to-use workflow environment, so that biomedical researchers can easily design their ‘discovery workflows’ and execute them securely on the grid.

A range of demonstrators, stemming from the user-defined scenarios, together with these core set of components will enable us to both begin evaluation and gather additional and more concrete requirements from our users. These will allow us to improve and refine the facilities of the ACGT services.

#### Community involvement

ACGT’s vision is to become a pan-European voluntary network connecting individuals and institutions to enable the sharing of data and tools and thereby creating a European-wide Web of cancer clinical research. The project promotes the principle of open source and open access, thus enabling the gradual creation of a European Biomedical Grid on Cancer. Hence, the project plans to introduce additional clinical trials during its lifecycle.

It is our strong belief that the project will not fully attain its objectives unless it succeeds in attracting the involvement of the user communities, that is clinical and research centres active in post-genomic cancer research.

It is, therefore, our objective to devise mechanisms to allow the community to engage in a bio-directional dialogue with us. Currently, the project website (www.eu-acgt.org) provides a limited set of such mechanisms, which will be extended and enriched as the project matures. We invite the clinical research community on cancer to join us in the effort to realize the vision that lies behind the project, that is automation of the biomedical knowledge discovery process.
